# Radiation recall pneumonitis induced by PD-1/PD-L1 blockades: mechanisms and therapeutic implications

**DOI:** 10.1186/s12916-020-01718-3

**Published:** 2020-09-18

**Authors:** Feifei Teng, Min Li, Jinming Yu

**Affiliations:** 1 Department of Radiation Oncology, Shandong Cancer Hospital and Institute, Shandong First Medical University and Shandong Academy of Medical Sciences, 440 Jiyan Road, Jinan, China; 2 Department of Surgery, Department of Medicine, The University of Oklahoma Health Sciences Center, Oklahoma City, OK USA

**Keywords:** Radiation recall pneumonitis, Radiation, Anti-PD-1/PD-L1, Lung cancer

## Abstract

**Background:**

The synergistic effect of radiotherapy (RT) in combination with immunotherapy has been shown in several clinical trials and case reports. The overlapping pulmonary toxicity induced by thoracic RT and programmed death 1/programmed death ligand-1 (PD-1/PD-L1) blockades is an important issue of clinical investigation in combination treatment. Thus far, the underlying mechanism of this toxicity remains largely unknown.

**Main text:**

In this review, we discuss the unique pattern of radiation recall pneumonitis (RRP) induced by PD-1 blockade. The clinical presentation is different from common radiation pneumonitis (RP) or RRP induced by cytotoxic drugs. The immune checkpoint inhibitors may evoke an inflammatory reaction in patients’ previously irradiated fields, with infiltrating lymphocytes and potential involvement of related cytokines. All RRP patients have showed durable response to anti-PD-1/PD-L1. RRP is manageable; however, interruption of checkpoint blockades is necessary and immunosuppressive treatment should be started immediately. Further analyses of the predictive factors, including RT dosimetric parameters, tumor-infiltrating lymphocytes (TILs), and PD-L1 expression, are needed given the wide use of immune checkpoint inhibitors and high mortality from lung toxicity with the combination treatment.

**Conclusion:**

Immune checkpoint inhibitors may evoke an RRP in the patients’ previously irradiated fields. Interactions between immune checkpoint inhibitors and radiotherapy should be studied further.

## Background

Programmed death 1 (PD-1) and programmed death ligand-1 (PD-L1) blockades have shown clinical activity and marked efficacy in the treatment of advanced non-small cell lung cancer (NSCLC). Several PD-1/PD-L1 blockades have been approved by the Food and Drug Administration (FDA) and the European Agency of Medicine (EAM) in the treatment of NSCLC [1–7]. Pembrolizumab has been approved as first-line treatment for advanced squamous or non-squamous NSCLC with PD-L1 expression ≥ 50% and as second-line treatment for advanced squamous or non-squamous NSCLC with PD-L1 expression ≥ 1% [8, 9]. The use of nivolumab and atezolizumab has been approved for advanced squamous or non-squamous NSCLC, independent of PD-L1 status, after at least one previous chemotherapy regimen [2, 10–12]. Durvalumab has been approved as consolidation therapy after chemo-radiotherapy in unresectable stage III NSCLC [13].

The synergistic effect of radiotherapy (RT) in combination with immunotherapy has been reported in several case reports and clinical trials [14, 15]. Since the potential pulmonary toxicity induced by thoracic RT and PD-1/PD-L1 blockades could overlap, pneumonitis is an important point of clinical investigation in combination treatment. Thus far, the nature of this toxicity remains largely unknown. Herein, we discussed the unique pattern of radiation recall pneumonitis (RRP) induced by PD-1 blockade. With the dramatic increase in checkpoint immunotherapy usage, this new pattern of immunotherapy-related toxicity merits increased awareness with a focus on the clinical characteristics, underlying mechanisms, and management strategies.

## Main text

### Clinical and patients characteristics

Based on the previous trials and meta-analysis, all-grade and grade 3–4 pneumonitis occurred in 3–5% and 1%, respectively, of patients with NSCLC who received PD-1/PD-L1 blockades [10, 16, 17]. The incidence of pneumonitis may be higher when combined with RT, but the clinical data were limited. Louvel et al. reported two cases of pneumonitis in six patients who received concomitant PD-1/PD-L1 blockades with SBRT [18]. In a secondary analysis of the KEYNOTE-001 trial, which studied the use of pembrolizumab for patients with advanced NSCLC, all-grade pneumonitis occurred more frequently in patients who received previous thoracic RT than in those with no previous thoracic RT (63% vs. 40%) [19]. In the phase 2 randomized PEMBRO-RT trial, 92 patients were randomized to receive pembrolizumab either alone or after radiotherapy (3 fractions of 8 Gy) to a single tumor site. Pneumonitis occurred more often in the pembrolizumab combined with radiotherapy group than in the control group (26% vs. 8%) [15].

RRP is characterized by an inflammatory reaction within the previously treated radiation field after administration of specific treatment [20–22]. Most RRP reported previously was induced by chemotherapy, such as gemcitabine and taxanes. Immunotherapy-induced RRP was rarely reported and showed some differences from RRP induced by chemotherapy. First, according to previous literature [23, 24], the interval between the end of radiotherapy and diagnosis of immunotherapy-induced RRP could be nearly 2 years [23]. The corresponding intervals for RRP induced by chemotherapy ranged from 71 to 202 days [21]. Second, the patients with immunotherapy-induced RRP often had durable response to PD-1/PD-L1 blockades. In the two RRP cases reported by Shibaki et al., the corresponding intervals were 660 and 664 days; both of the cases showed a durable response [14]. In the study of Eze et al., all 3 patients achieved a durable response [15]. Although we cannot draw definitive conclusions based on the limited data [14, 15], this finding indicated that the occurrence of RRP might be related to favorable response to PD-1/PD-L1 blockade immunotherapy. However, chemo-induced RRP was not found to be related to the therapeutic effect of chemotherapy [20, 21, 25].

Age > 70 years and prior interstitial lung disease were reportedly associated with higher incidence of immunotherapy-related pneumonitis [26]. In a study conducted by Suresh et al., the risk of pneumonitis was higher for males than females, 0.25 vs. 0.19 per year [27]. In contrast, smoking status was not a risk factor in these patients [28]. In the above studies, the histology of squamous carcinoma was more common in patients with immunotherapy-related pneumonitis [29]. In addition, the absence of extrathoracic metastases was associated with increased incidence of immunotherapy-related pneumonitis [26].

A study of 148 lung cancer patients who received definitive chemoradiation was reviewed to identify factors that may predict severe radiation pneumonitis (RP) [30]. The most significant factor for predicting RP was performance status. The incidence of severe RP was 16% for PS-1 patients and 2% for PS-0 patients, respectively. In addition, females were more likely to develop severe RP than males. Movsas et al. analyzed sociodemographic factors of 1450 patients treated in nine prospective RTOG trials [31]. They found that lower lobe primaries, presence of family with cancer, married relationship, and the interaction of female sex with low KPS status were associated with grade > 3 radiation pneumonitis. A history of smoking was another potential factor predictive of RP [32]. Previous radiation history was also associated with higher incidence of RP [33]. In a pooled analysis, 88 studies were included to study the risk factors for pneumonitis after RT of the thorax [34]. Patient age and tumor size were significantly associated with rate of grade 2 or greater RP. However, histology, GTV, PTV, and tumor location (central versus peripheral) were not significant.

Considering the indicators for immunotherapy-related pneumonitis and radiation pneumonitis, older patients with large tumor size, low KPS status, and prior interstitial lung disease should be cautious about the occurrence of RRP.

### Therapy regimens

Based on previous studies, immunotherapy was often delivered after thoracic RT. The interval between thoracic RT and immunotherapy varied from 1 day to 11.5 months, and the incidence of pneumonitis was between 0 and 17% [13, 15, 19]. It seemed there were no relationships between the rate of pneumonitis and the time intervals between RT and anti-PD-1/PD-L1 treatment, but further studies are needed to clarify the associations between pneumonitis and therapy regimens that are delivered sequentially and concomitantly.

A higher incidence of pneumonitis was reported with the use of PD-1 inhibitors compared with PD-L1 inhibitors and in treatment-naïve patients [16]. The PD-1/PD-L2 interactions with PD-1 inhibitors might be the reason for the higher incidence of pneumonitis in PD-1 inhibitors. Preclinical experiments suggested that PD-1 blockades may increase PD-L2 availability for binding to repulsive guidance molecule b (RGMb), which could lead to pneumonitis [35].

### RT dosimetric factors

RT dose and fractionation, which often affect clinical management decisions with respect to tumor control and lung tolerance, are important. Dosimetric parameters have been widely assessed as predictors for the development of radiation pneumonitis. In a study conducted by Jenkins and Watts, dosimetric parameters, pulmonary function, and clinical parameters were analyzed in patients who received RT at the same dose and with an identical technique for NSCLC. The fractional volume of lung receiving > 5–20 Gy, mean lung dose (MLD), absolute volume of lung spared from receiving > 5–15 Gy, craniocaudal position of the isocenter, total lung capacity, and transfer coefficient for carbon monoxide (KCOc) were significantly correlated with the risk of pneumonitis [36]. As reported, MLD of 15 Gy, 17.5 to 20 Gy, 22.5 to 25 Gy, and 27.5 Gy resulted in 0%, 13%, 21%, and 43% incidence of all grades of RP, respectively [37]. In a meta-analysis, MLD and the percent volume of the total lung receiving a dose greater than 20 Gy (V20) were significant factors for higher risk of grade 2 or greater RP. Another study demonstrated that MLD from total lung excluding-plan target volume (Lung-PTV) may be more accurate and promising to predict acute symptomatic radiation pneumonitis in intensity-modulated radiation therapy (IMRT)-treated lung cancer patients [38]. With the development of radiation equipment, IMRT and stereotactic body radiation therapy (SBRT) have been widely used in the treatment of lung cancer. The incidence of grade > 3 RP was lower when using IMRT than when using 3-dimensional techniques (3DCRT), since IMRT could allow for extensive reductions of radiation dose to normal lung tissues [39]. MLD and the volume of the total lung receiving a dose greater than 5, 7, and 10 Gy were also associated with RP for SBRT [40]. In addition, proton beam therapy has been increasingly used for the treatment of lung cancer and may further reduce radiation dose to normal lung tissues and reduce the risk of RP [41]. As for immunotherapy-related pneumonitis, in a retrospective study, the relationship between chest-RT and development of immune-related pneumonitis in NSCLC patients treated with anti-PD-1/PD-L1 was analyzed. However, no RT parameter was significantly associated with pneumonitis [42]. Since RP is correlated with the dose delivered to a particular fractional volume of the lung, RT dose and fractionation, especially MLD, deserve attention and potential pneumonitis should be monitored in patients receiving combination treatment.

### Radiographic patterns

In the majority of RRP cases, the area of pneumonitis matched the irradiated area. The most common radiographic pattern of RRP on chest CT was the cryptogenic organizing pneumonia (COP) pattern, followed by the non-specific interstitial pneumonia (NSIP) pattern, the hypersensitivity pneumonitis (HP) pattern, and the acute interstitial pneumonia (AIP)/acute respiratory distress syndrome (ARDS) pattern. The radiographic patterns were associated with volume of irradiated area and the toxicity grades of RRP. AIP/ARDS was correlated with the highest grade (grade 3), then the COP pattern as grade 2, and the HP and NSIP patterns had the lowest grade 1. Ground glass opacities (GGO), reticular opacities, and consolidations were observed in some cases of RRP.

### Underlying mechanisms

Thoracic radiotoxicities can produce both acute and long-term lung toxicities that occur months to years after treatment. Around 15% of patients experience pneumonitis within 2 to 3 months after thoracic RT [43, 44]. Pulmonary fibrosis can be considered a recovery of lung injury after radiation. The gamma delta T cells (γδT) cells help prevent progression of fibrosis and suppress CD4^+^ cell recruitment [45]. Several groups have found abundant lymphocytes infiltrating the lung tissues and elevated CD4/CD8 ratios in bronchoalveolar lavage fluid from patients with radiation pneumonitis [43, 46–50]. In addition, the T helper type 22 (TH22) and follicular helper T (TFH) cells may have possible effects on the host defense against bacteria and viruses in the lung [51, 52]. Previously, it was assumed that immunotherapy via PD-1/PD-L1 blockade works by reinvigorating pre-existing exhausted tumor-infiltrating lymphocytes (TILs). Recently, it was indicated that the majority of tumor-specific TILs after immunotherapy have T cell receptors (TCRs) that were not identified pre-treatment, suggesting that the TILs are newly recruited post-therapy [53]. A dynamic monitoring of the changes of the specific T cell populations and phenotypes during immunotherapy in previous irradiated models could help monitoring the RRP and the anti-tumor response.

Transforming growth factor β (TGF-β) plays a part in progression of radiation-related fibrosis [54]. Rube and Chen et al. observed that the serum levels of TGF-β and IL-6 were elevated prior to and after radiotherapy and correlated with radiation-induced pneumonitis [55, 56]. Tumor necrosis factor (TNF)-α leads to TGF-β1 induction and is known to contribute to fibrosis development [57]. Hence, it has become a target molecule to monitor the progression of fibrosis. The cytotoxic actions of TNF also play an important role in anti-PD-L1 treatment. Nuclear factor–kB (NF-kB) signaling is crucial in regulating the production of TNF-α, which was associated with the function of cytotoxic T lymphocytes and maturation of dendritic cells in the use of PD-1/PD-L1 blockades for cancer treatment [58]. Elevated TNF was observed with a combination treatment of anti-PD-L1 and radiation [59]. The potential synergy may also activate inflammation-mediated injury.

In addition, interleukins (IL)-4, IL-6, IL-10, IL-13, IL-17, and IL-18 are demonstrated to be associated with radiation-related pneumonitis and the therapeutic effects of anti-PD-1/PD-L1 [60]. Buttner et al. observed increased IL-4 gene transcription and synthesis in lung tissues after radiation, and substantial IL-4 secretion by macrophages in radiation-induced fibrosis [61]. Wilson et al. demonstrated that IL-17A gene knockout could decrease the severity of lung injury in mice, demonstrating the important role of IL-17A in fibrosis and inflammation [62]. IL-4 and IL-13 were also shown to facilitate fibrosis by promoting TGF-β [63]. This finding may explain the associations between RRP and response to anti-PD-1/PD-L1 in our case and previous reports. The cytokines resulting in pneumonitis also play a pivotal role in PD-1/PD-L1 blockade immunotherapy.

**Fig. 1:**
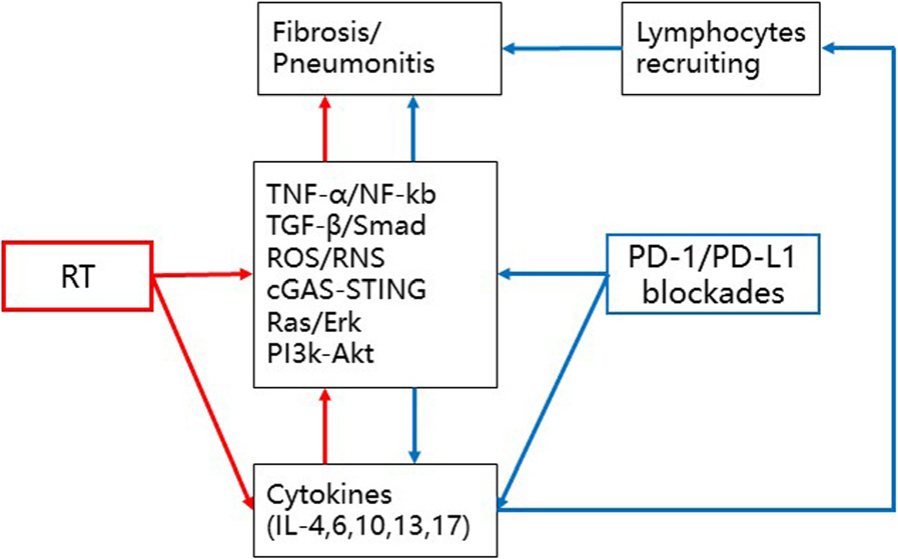
Cytokines and relative signaling pathways potentially involved in RRP. Tumor necrosis factor-α (TNF-α); transforming growth factor β (TGF-β); interleukins 4, 6, 10, 13, 17, and 18 (IL-4, 6, 10, 13, 17, 18); myeloid differentiation primary response 88 (MyD88); cGMP–AMP synthase (cGAS)–stimulator of interferon genes (STING); nuclear factor kappa-light-chain-enhancer of activated B cells (NF-kB); reactive oxygen species/reactive nitrogen species (ROS/RNS); extracellular regulated protein kinases (Erk); and phosphatidylinositol 3-kinase (PI3K)

**Fig. 2:**
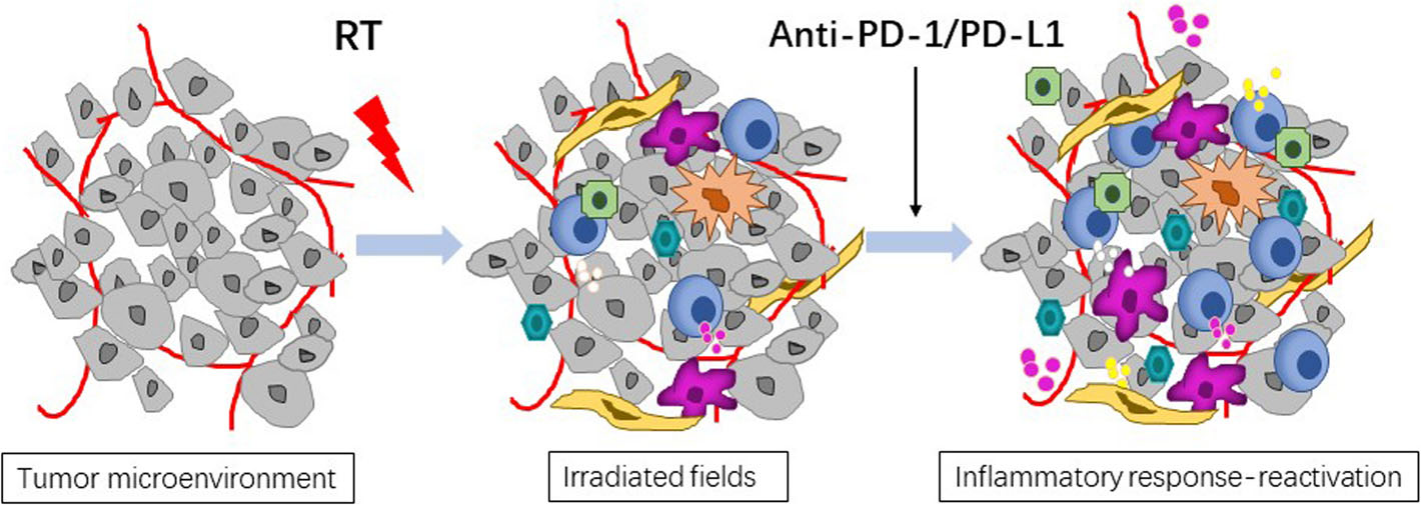
Immune mechanisms of RRP triggered by anti-PD-1/PD-L1. The immune checkpoint inhibitors evoke an inflammatory reaction in previously irradiated fields

Brickey et al. investigated the role of myeloid differentiation primary response 88 (MyD88) in regulating nuclear factor kappa-B (NF-kB) activating responses and innate immunity in post-radiation lung tissues. They found that MyD88 was instrumental for regulating inflammatory processes that aid in recovery from radiation [55, 64]. The activation of cGMP–AMP synthase–stimulator of interferon genes (cGAS-STING) signaling and ROS/RNS also plays an important role in lung injury mediated by non-infectious inflammatory processes [65, 66]. The major signaling pathways involved in immunotherapy-induced RRP are shown in Fig. 1. The activations and interactions of these inflammatory-related signals also contributed to the therapeutic effects of anti-PD-1/PD-L1, with or without radiotherapy. Anti-PD-1/PD-L1 therapy may have evoked an inflammatory reaction mediated by the lymphocytes, cytokines, and proteins as a result of radiation exposure in patients’ previously irradiated fields (Fig. 2).

### Immunologic factors

Understanding the molecular and cellular processes of RRP helps in finding biomarkers that predict the risk for developing radiation pneumonitis during checkpoint immunotherapy. Tumor-infiltrating lymphocytes (TILs) were strongly associated with response to anti-PD-1/PD-L1 immunotherapies [67]. Since increased rates of lymphocytes, macrophage, and neutrophils were found in bronchoalveolar lavage (BAL) fluid from the majority of RPP patients, high TILs are most likely associated with RRP. However, the predictive effect was limited by the invasive operation. A number of researchers have reported the predictive effects of circulating lymphocytes [68–70]. The percentage of circulating B cells and T lymphocytes may reflect the body’s immune status and may be a potential therapeutic target for the treatment of pneumonitis [71]. Further, circulating and lung Th17 cells contributed to pulmonary fibrosis and inflammation [72]. An imbalance of circulating Th17 cells and regulatory T (Treg) cells was associated with the deterioration of pulmonary injury [73].

Transforming growth factor α (TGF-α), a protein coded by the TGFA gene, was a potential biomarker for RRP. TGF-α was able to induce lung fibroblasts to myofibroblasts and to stimulate collagen synthesis [74, 75]. Plasma TGF-α levels could be used to stratify patients into groups at low, intermediate, and high risk to develop RP [76, 77]. Monitoring plasma TGF-α levels may be useful to predict RRP during the course of PD-1/PD-L1 blockades.

IL-6 is an important cytokine responsible for inflammation and RRP development [78]. It is synthesized and secreted by various lung parenchyma cells and regulates inflammatory and immune responses [79]. Hence, levels of serum IL-6 could be used to estimate the inflammatory status of the lung. However, immunotherapy could also increase the level of IL-6, especially in good responders, which may disturb its predictive effects [80].

As a pivotal molecular target during treatment of anti-PD-1/PD-L1, PD-L1 expression has been of concern. Expression of PD-1/PD-L1 in lung tissue may be related to immune-related pneumonitis. With limited data, we cannot draw conclusions about the association of PD-1 or PD-L1 expression with pneumonitis. Since the host immune status plays an important role in both therapeutic effects and lung injury with radiation combined with anti-PD-1/PD-L1, further studies are needed to evaluate its predictive effect.

### Management strategies

Steroids and corticosteroids have been widely used in different inflammatory diseases, such as radiation pneumonitis and some autoimmune diseases. Prednisone, which could improve lung function and minimize lung tissue toxicity and symptoms of radiation-induced pneumonitis, was the most commonly used drug [81]. In grade 1 to 2 pneumonitis, treatment consists of oral steroids with prednisone 1 mg/kg/day or equivalent. Steroids should be tapered over 4 to 6 weeks after recovery, and rechallenge of immunotherapy should be delayed until the dose of steroids equals 10 mg of oral prednisone per day or less. In grade 3 to 4 pneumonitis, treatment should consist of high-dose i.v. corticosteroids with prednisolone 2–4 mg/kg daily or equivalent and immunotherapy should be discontinued permanently. If there is no improvement in patient’s condition or imaging, additional immunosuppressive strategies should be implemented. Tapering of steroids should be careful and slow, over 6 weeks or more [82]. Also, concurrent broad-spectrum antibiotics are recommended because of the potential for overlapping presentation and infection [83, 84].

In addition to steroids and corticosteroids as the mainstay of treatment, angiotensin-converting enzyme inhibitor could potentially reduce the risk of symptomatic radiation pneumonitis in male patients and patients receiving low MLD with NSCLC [85, 86]. Oxygen therapy was suggested as a supportive therapy. It could relieve breathlessness by increasing the oxygen in blood and in tissues. Further, some molecular approaches targeting the intermediates involved in the development of inflammatory response in the lungs, such as DNA intercalator and anti-TGF-β type 1 receptor, were studied in preclinical practice [87, 88].

## Conclusions

In conclusion, RRP induced by PD-1 blockade is the unique pattern of radiation-related toxicity. The clinical presentation was different from common RP and RRP induced by cytotoxic drugs. The immune checkpoint inhibitors may have evoked an inflammatory reaction in patients’ previously irradiated fields, with potential involvement of infiltrating lymphocytes and related cytokines. All RRP patients showed durable response to anti-PD-1/PD-L1. It was manageable, and sufficient steroids or corticosteroids are needed. A further analysis of the predictive factors is needed with the wide use of immune checkpoint inhibitors and high mortality of this kind of lung toxicity with combination treatment.

## Data Availability

All data generated or analyzed during this study are included in this published article.

## Abbreviations

AIP: Acute interstitial pneumonia
ARDS: Acute respiratory distress syndrome
BAL: Broncho-alveolar lavage
COP: Cryptogenic organizing pneumonia
COPD: Chronic obstructive pulmonary disease
CTCs: Circulating tumor cells
HP: Hypersensitivity pneumonitis
IL-4, 6, 10, 13, 17, 18: Interleukins 4, 6, 10, 13, 17, 18
KCOc: Carbon monoxide
MLD: Mean lung dose
Myd88: Myeloid differentiation primary response 88
NF-kb: Nuclear factor kappa-light-chain-enhancer of activated B cells
NSCLC: Non-small cell lung cancer
NSIP: Non-specific interstitial pneumonia
PD-1: Programmed death 1
PD-L1: Programmed death ligand-1
PTV: Plan target volume
RGMB: Repulsive guidance molecule b
ROS/RNS: Reactive oxygen species/reactive nitrogen species
RRP: Radiation recall pneumonitis
RT: Radiotherapy
cGAS-STING: Cgmp–AMP synthase–stimulator of interferon genes
TFH: Follicular helper T cell
TGF-β: Transforming growth factor β
TH22: T helper type 22
TILs: Tumor-infiltrating lymphocytes
TNF-α: Tumor necrosis factor-α
Treg: Regulatory T
γδT: Gamma delta T cells
